# Using Aggregation-Induced Emission to Understand Dipeptide Gels

**DOI:** 10.3390/gels4010017

**Published:** 2018-02-09

**Authors:** Ana M. Castilla, Bart Dietrich, Dave J. Adams

**Affiliations:** 1 Department of Chemistry, University of Liverpool, Crown Street, Liverpool L69 7ZD, UK; a.m.castilla@qmul.ac.uk; 2 School of Chemistry, University of Glasgow, Glasgow G12 8QQ, UK; bart.dietrich@glasgow.ac.uk

**Keywords:** hydrogel, self-assembly, supramolecular, aggregation, fluorescence

## Abstract

We describe the formation of structured liquids and gels from a functionalised dipeptide based on tetraphenylethylene. Tetraphenylethylene is well-known to be able to undergo aggregation-induced emission. We use the emission data to understand the behaviour of the dipeptide in water under a variety of conditions. The dipeptide forms viscous solutions at high pH. Gels can be formed by a pH-trigger, but syneresis occurs. Addition of a calcium salt also leads to a gel with slight syneresis. Addition of sodium chloride leads to a self-supporting material, but this is not a true gel from the rheological perspective. From the emission data, we infer that there are limited structural changes on addition of sodium chloride or acid, but there are significant changes in molecular packing when the gel is formed by addition of a calcium salt.

## 1. Introduction

A range of *N*-functionalised amino acids and dipeptides can be used to form hydrogels [1]. These are gaining significant interest and have been used for a range of different applications, such as cell culturing and differentiation, drug delivery, responsive materials, mineralization, and to prepare functional surfaces [1,2]. Gels with a range of mechanical and material properties can be prepared depending on the gelator structure, and critically, the method used to trigger gel formation. For example, gels can be formed from the same gelator that exhibit very different recovery after shear. Since it is often difficult to predict in advance whether a molecule will be an effective gelator [3], it is useful to be able to control the properties of a gel formed by one gelator as opposed to exhaustively screen different gelators that give a gel with the desired properties.

The gels are formed by the self-assembly of the gelator molecules into fibrous structures that entangle to form a network [4]. The underlying microstructure, i.e., how the fibres are distributed in space, can be very different depending on how the gels are formed. For example, when a solvent-trigger is utilized [5], whereby the gelator is initially dissolved in a good solvent such as DMSO before water is added, spherulitic domains are usually formed [4,6]. More homogenous networks are often formed with a slow pH trigger or by addition of a salt [4]. A key question is how the molecules are packed in the self-assembled structures. A model has been suggested for one example, Fmoc-diphenylalanine [7], although it is not clear if this can be applied to other examples, and there are other hypotheses [8] even for this gelator [9]. Most of the techniques that can provide information on the molecular packing in the gels require drying of the sample. This process can lead to artefacts in the measurements [10] and therefore it is of interest to find new techniques that can probe the molecular packing in situ.

Aggregation most commonly leads to self-quenching effects and, hence, to a decrease in the emission from the molecule. In contrast, aggregation-induced emission (AIE) is a phenomenon whereby aggregation results in an increase in the emission [11,12]. Molecules that exhibit AIE tend to be those where rotations provide relaxation pathways of excited states in the solution state, but aggregation results in restrictions to those molecular rotations, blocking those fast deactivation pathways and fluorescence occurring instead. There are a number of examples where gelators bearing moieties able to exhibit AIE have been used. Gelation occurs by the formation of self-assembled structures, and this process can thus lead to AIE. Specific relevant examples to this work include those where tetraphenylethylene (TPE, the AIE chromophore) has been coupled to dipeptides [13,14,15], or oligopeptides [16,17]. 

Here, we describe a TPE-based gelator, whereby diphenylalanine is coupled to a core that is capable of AIE. This gelator self-assembles in water and gels can be formed. We show how AIE can be used to develop a deeper understanding of the self-assembly process this gelator undergoes during gel formation. This provides insights into the assembly of *N*-functionalised dipeptides in water.

## 2. Results and Discussion

### 2.1. Gelation Studies

**Figure 1:**
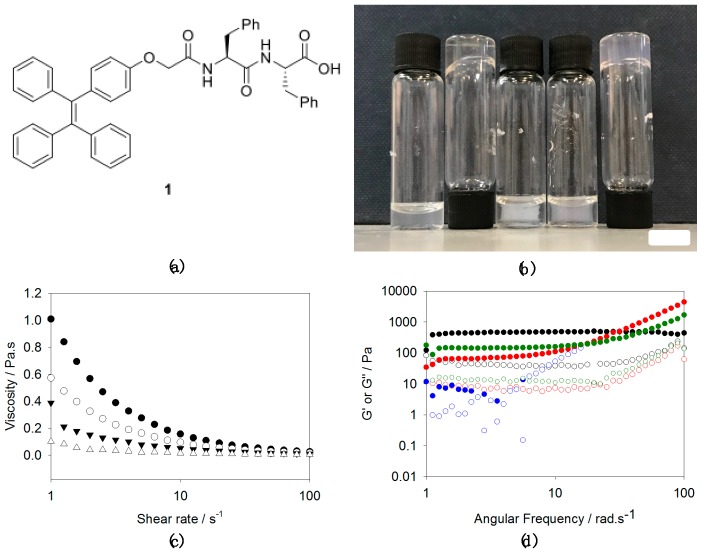
(**a**) The structure of gelator **1**; (**b**) from left to right: a solution of **1** at 5 mg/mL at high pH; a solution of **1** at 10 mg/mL after a heat-cool cycle, a solution of **1** at 5 mg/mL after the addition of GdL (5 mg/mL), a solution of **1** at 5 mg/mL after the addition of CaCl_2_, and a solution of **1** at 5 mg/mL after the addition of NaCl (18 mg/mL). Syneresis can be seen in the GdL-triggered and Ca-triggered gels. The scale bar represents 1 cm; (**c**) viscosity data for solutions of **1** at different concentrations at pH 10.5 (5 mg/mL (●); 3.75 mg/mL (○); 2.5 mg/mL (▼); 1.25 mg/mL (Δ)); (**d**) frequency sweeps for self-supporting samples formed by the addition of NaCl to solutions of **1** (black data 10 mg/mL; green data 7.5 mg/mL; red data 5 mg/mL; blue data 2.5 mg/mL; in all cases, full symbols represent G′ and open symbols represent G″ and a constant ratio of NaCl: **1** of 1:8 was used).

The gelator (**1**, Figure 1a) used in the study was synthesised from 2-[4-(1,2,2-triphenylethenyl)phenoxy]acetic acid, which is based around TPE, a well-known core that undergoes aggregation-induced emission. To this, we coupled diphenylalanine ethyl ester. Deprotection of the ethyl group from the *C*-terminus of the dipeptide afforded gelator **1** in good yield (70% overall).

Transparent solutions of **1** in water at high pH (typically pH 10.5 to 11) were obtained by the addition of one molar equivalent of sodium hydroxide to a dispersion of **1** (Figure 1b). This is consistent with our previous work on similar molecules [18], as well as for a closely related TPE [14]. We note, however, that Sun et al. were unable to solubilise a closely related analogue by adjusting the pH [13]. The solutions of **1** at high pH were noticeably viscous above 1 mg/mL, with the viscosity being concentration dependent (Figure 1c). These data are consistent with **1** forming worm-like micelles under these conditions, similarly to other very hydrophobic *N*-functionalised dipeptides [18,19]. In a similar manner to our recent results with a naphthalene-diphenylalanine [18], heating and cooling the solution at a concentration of 10 mg/mL resulted in a significantly more viscous solution, which was stable to vial inversion (Figure 1b). An apparent increase in viscosity was also observed at 5 mg/mL, but this sample was not stable to inversion.

By analogy with our other work on *N*-functionalised dipeptides, [4] we attempted to form gels using different types of trigger. First, we added glucono-δ-lactone (GdL), which hydrolyses to gluconic acid to bring about a slow, uniform pH change [20,21]. For solutions of **1**, this slow acidification leads to the formation of a gel, but with significant syneresis (Figure 1b). Similarly to related materials [22], this syneresis seems to be inherent to the material such that it was impossible to find conditions where this did not occur. For this kind of gelator that forms worm-like micelles at high pH, gelation can often also be induced by the addition of a divalent cation such as calcium [18]. On addition of a solution of calcium chloride to a solution of **1** at high pH, a gel was initially formed, but again syneresis occurred over time; this means that the sample is not self-supporting, but a gel can be seen surrounded by liquid (Figure 1b). In contrast, addition of sodium chloride results in the formation of a self-supporting material (Figure 1b). For a constant ratio of NaCl to **1**, at low concentrations of **1**, rheologically these are not true gels, as there is a significant frequency dependence (Figure 1d). The plateau modulus below 1 rad/s increases with the concentration of **1** used, such that frequency independent materials are formed at 10 mg/mL. Strain sweeps (Figure S1, Supporting Information) show that the frequency sweeps were carried out in the LVE region for the higher concentrations of **1**. At lower concentrations, the materials can again be seen to not be true gels. At all concentrations, the materials only break down at relatively high strains. We ascribe the formation of the material that can be stable to inversion to charge screening by the addition of sodium chloride, allowing the worm-like micelles to entangle to a greater extent.

### 2.2. AIE Studies

**Figure 2:**
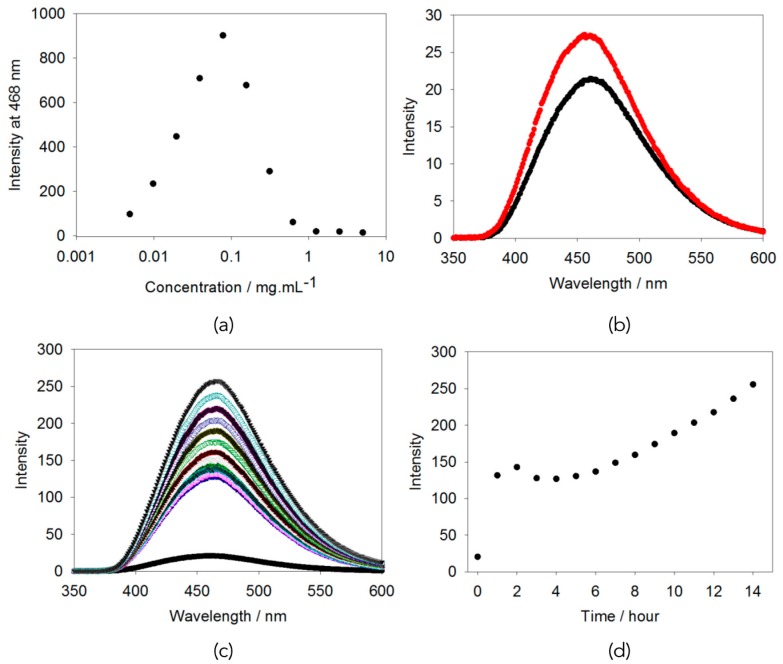
(**a**) Fluorescence intensity at 468 nm (λ_ex_ = 330 nm) for a dilution series of **1** at pH 10.5; (**b**) fluorescence spectrum for a solution of **1** at a concentration of 5 mg/mL and a pH of 10.5 before (black) and after (red) a heat-cool cycle; (**c**) changes in fluorescence on addition of a solution of calcium chloride to a solution of **1** at 5 mg/mL (the colors are to provide distinction between runs, the intensity increases with time as can be seen in (**d**)); (**d**) Change in emission at 468 nm with time on addition of a solution of calcium chloride to a solution of **1** at 5 mg/mL.

To understand this behaviour in more detail, we examined the propensity for the tetraphenylethylene to exhibit aggregation induced emission (AIE). For similar molecules, gelation has been reported to be accompanied by an increase in emission intensity, although in those cases, gelation has generally been achieved by initially fully dissolving the gelator in a good organic solvent, before adding water as an anti-solvent [13,14]. Here, it is clear that there is significant aggregation at high pH, as expected from the viscosity data above. Plotting the emission intensity against the concentration, it is clear that 5 mg/mL is already in the self-quenched regime (Figure 2a) [12]. Hence, the data appear entirely consistent with a typical molecule undergoing aggregation induced quenching [12]. It could be that the packing within the aggregated structures does not effectively restrict rotation of the TPE core. We note that where AIE has been reported previously for a TPE-dipeptide on addition of a mercury salt, the concentration used was 3 × 10^−6^ M [15], whilst here, at 5 mg/mL, we are at around 7 × 10^−4^ M.

Choosing a concentration of 5 mg/mL for the pre-gelation solution, as this has a reasonable emission and gel form, we examined the effect of the different assembly and gelation methods on the fluorescence. First, the fluorescence from a solution before and after heating were compared. The data show that there is very little change in intensity (Figure 2b; note the typical errors between repeat samples are 2 a.u.) implying that the molecules are in a very similar environment before and after the heat-cool cycle. We previously showed that for a related gelator, the structures leading to this viscosity were best explained by being hollow cylinders [18]. The heat-cool cycle led to a shrinking in the diameter of both the cylinder and the core. This led to a lengthening of the structures and hence an increase in the viscosity. Assuming that a similar process is occurring here, it seems that locally there are few changes, and hence the fluorescence is only slightly affected, although there is a small AIE effect. We note that there are no significant changes in the UV-Vis spectra when the sample has been heated and cooled (see Figure S2, Supporting Information).

**Figure 3:**
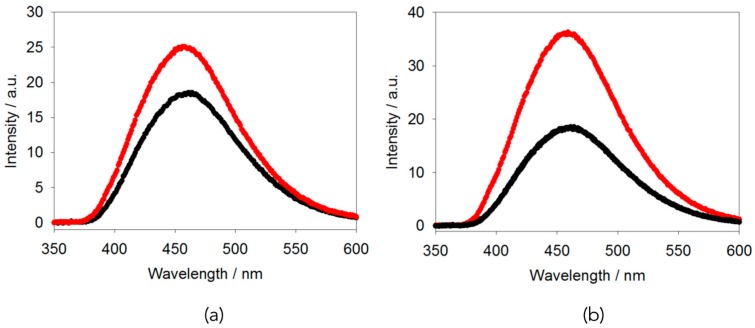
(**a**) Fluorescence spectrum for a solution of **1** at a concentration of 5 mg/mL and a pH of 10.5 before (black) and 7 h after the addition of GdL (red); (**b**) fluorescence spectrum for a solution of **1** at a concentration of 5 mg/mL and a pH of 10.5 before (black) and after (red) the addition of NaCl.

Similarly, adding an aliquot of a solution of calcium chloride to a solution of **1** results in the fluorescence increasing dramatically with time (Figure 2c). The increase in peak intensity is initially sharp, followed by a slight decrease and then slow increase (Figure 2d). We ascribe this to the method used to add the calcium solution resulting in slow diffusion down the sample. In comparison, adding a solution of sodium chloride to a solution of **1** resulted in only a slight increase in the fluorescence intensity (Figure 3b). In these cases, there are slight changes in the UV-Vis spectra on addition of the calcium solution, arising from scattering from the syneresis; there is little change to the UV-Vis spectrum on adding sodium chloride (see Figure S2, Supporting Information).

Finally, adding GdL to a solution of **1** results in a slow pH change (again, note that typical errors between repeat samples are 2 a.u.). Following the fluorescence with time shows that there are again only slight increases in the fluorescence over about 7 h (Figure 3a). It is difficult to follow this process over a longer time, as the decrease in pH also results in syneresis (see above) and changes in turbidity (Figure S2, Supporting Information).

Putting this all together, the small increase in fluorescence for the gelation with GdL or sodium chloride is consistent with there being very little change on gelation in terms of the molecular packing. The small increase could be interpreted as a slight AIE effect, but is very low if this is the case. Conversely, the increase in intensity when the sample is gelled with a calcium salt is significant. This could be interpreted as being due to dilution (as suggested by Figure 2a). However, by comparison to the data shown in Figure 2a, this would require an effective dilution by a factor of approximately ten, which seems unlikely; there is slight syneresis during this assembly, and so we would instead expect, effectively, an increase in the local concentration of **1**, as this syneresis is thought to arise from lateral association of fibres [22]. As an alternative explanation, we could interpret this increase in fluorescence as being due to aggregation-induced emission. Gelation by the addition of a divalent salt is thought to occur by the binding of the metal ions by the carboxylates. Conceptually this could mean that there are little changes to the molecular packing, but this seems inherently unlikely and we have shown for related gelators that there are structural changes on addition of a calcium salt [19]. Hence, we suggest that in this case the fluorescence data imply that the binding to the calcium salts leads to a more rigid packing and hence significant AIE. 

## 3. Conclusions

In conclusion, the fluorescence data imply that for this molecule at high pH in water, aggregation-induced quenching occurs, as opposed to AIE. This is presumably due to the packing of the molecules in the self-assembled aggregates. Addition of acid results in a gel that undergoes syneresis, with a concomitant increase in fluorescence. Hence, it appears that AIE does occur on gelation. This is also observed for calcium-triggered gelation, with the AIE effect being significantly more pronounced in this case. Again however, slight syneresis occurs. Self-supporting translucent materials can be formed by the addition of sodium chloride to the solution of the TPE-gelator (**1**) at high pH. However, rheologically, there is some frequency dependence, meaning that these are not true gels, but presumably charge-screened worm-like micelles. Slight AIE was also observed on addition of sodium chloride. 

From these data, it appears that rotation of the TPE core is possible in the aggregates at high pH. This implies that the molecular packing is not highly restricted. On addition of a trigger however, AIE occurs which implies that the rotation is more restricted, with the effect being greatest for gelation triggered by the addition of the calcium salt. This would indicate that the molecular packing in the calcium chloride and sodium chloride triggered gels is different and again emphasizes that it is still unclear how the molecules are packed in these different self-assembled aggregates. 

## 4. Materials and Methods

The synthesis and characterization gelator **1** is described in full in the Supporting Information. 

### 4.1. Preparation of Solutions

As an example, to prepare a solution of **1** at 5 mg/mL, **1** (25 mg) was suspended in water (4.11 mL). An aliquot of a solution of sodium hydroxide (0.89 mL of a 0.1 M solution) was added. The mixture was stirred until a clear, viscous solution was formed. 

### 4.2. Acid-Triggered Gels

For the acid-triggered gels, typically a solution of **1** (1 mL) was added to a pre-weighed aliquot of glucono-δ-lactone (GdL) (8 mg). After swirling to dissolve the GdL, the sample was allowed to stand for 18 h.

### 4.3. Calcium-Triggered Gels

A solution of calcium chloride was prepared (200 mg/mL). An aliquot of this solution (60 μL) was carefully added to the top of a solution of **1** and gelation occurred as the solutions mixed by diffusion. 

### 4.4. Salt-Triggered Gels

Typically, a solution of NaCl in water (pH 11) was prepared at a concentration of 330 mg/mL. Aliquots of this solution were added to a solution of **1**. The sample was allowed to stand for 18 h before analysis.

### 4.5. Photographs

Photographs of the solutions and gels were taken on an iPhone7 (Apple, Cupertino, CA, USA).

### 4.6. Rheology

All rheological measurements were carried out on an Anton Paar Physica MCR 301 or 101 (Anton Paar, Nuremberg, Germany). Strain and frequency sweeps were performed using a vane and cup geometry. For this 2 mL of a gel was prepared as above directly in the 7 mL Sterlin vials used for the measurements. This ensured samples were not damaged and reproducible. All gels were measured in triplicate at 25 °C. Strain sweeps were performed between 0.1–1000% strain at a constant frequency of 10 rad/s, measuring 10 points per decade. Frequency sweeps were performed between 1–100 rad/s at a constant strain of 0.5%, measuring 10 points per decade.

### 4.7. Fluorescence Spectroscopy

Fluorescence spectra were collected on an Agilent Technologies Cary Eclipse Fluorescence Spectrophotometer (Agilent, ‎Santa Clara, CA, USA) in a 1 cm pathlength poly(methylmethacrylate) (PMMA) fluorescence cuvette. Spectra were collected using an excitation of 330 nm from 350–600 nm with a slit width of 5 nm. Samples were prepared as stated above but in the cuvettes.

### 4.8. UV-Vis Spectroscopy

Spectra were collected on an Agilent Technologies Cary 60 spectrometer in 0.1 mm path length quartz sandwich cuvettes. The sample was removed from the solution by pipette or from the gel by spatula and sandwiched into the cuvette. The resulted in the bulk structure being destroyed, but we assume not changing the local structure significantly.

## Supplementary Materials

The following are available online at http://www.mdpi.com/2310-2861/4/1/17/s1, a full description of the synthesis of **1** and all precursors. Figure S1: Strain sweeps for self-supporting samples formed by the addition of NaCl to solutions of **1** (a) 10 mg/mL; (b) 7.5 mg/mL; (c) 5 mg/mL; (d) 2.5 mg/mL; in all cases, full symbols represent G′ and open symbols represent G′′ and a constant ratio of NaCl: **1** of 1:8 was used, Figure S2: UV-Vis spectra for solutions of **1** at 5 mg/mL. Black data are for the as-prepared solution. Dark pink data are for a solution after heating and cooling. Red data are for a solution to which CaCl_2_ has been added. Green data are for a solution to which NaCl has been added. Blue data are for a solution to which GdL has been added. All data were collected in a 0.1 mm cuvette.
