# Supplementary material for: Using Aggregation-Induced Emission to Understand Dipeptide Gels
Source: Gels. 2018 Feb 9;4(1):17. doi: 10.3390/gels4010017 (PMC6318689; doi:10.3390/gels4010017)
Supplement: Supplementary file 1 [file gels-04-00017-s001.pdf]

Using aggregation-induced emission to understand dipeptide gels

Ana M. Castilla,[1] Bart Dietrich [2] and Dave J. Adams [2,*]

[1] Department of Chemistry, University of Liverpool, Crown Street, Liverpool, L69 7ZD, U.K.
[2] School of Chemistry, University of Glasgow, Glasgow, G12 8QQ, U.K.
* Correspondence: dave.adams@glasgow.ac.uk; Tel.: +44-0141-330-8672

Full synthetic details for the synthesis of 1 and all precursors.

4-(1,2,2-Triphenylethenyl)phenol (**1a**)

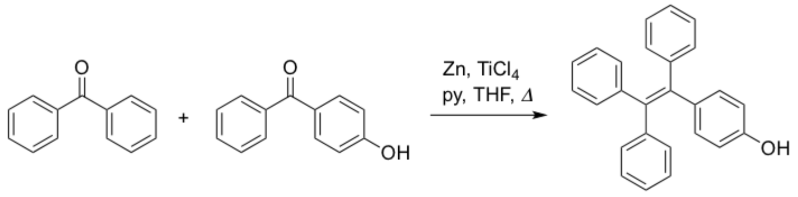

1a

The synthesis of **1a** was adapted from the literature.[1-2] Under inert atmosphere, a three-necked flask equipped with a magnetic stirrer was charged with zinc powder (8 g, 0.12 mol, 2.5 eq) and tetrahydrofuran (200 mL). The mixture was cooled to -5 °C, and titanium(IV) chloride (6.5 mL, 0.06 mol, 1.25 eq) was slowly added *via* syringe, keeping the temperature below 10 °C. The resulting suspension was warmed to room temperature and stirred for 0.5 h, then heated at reflux for 2.5 h. The mixture was again cooled to between -5 to 0 °C, pyridine (2.5 mL, 0.03 mol, 0.6 eq) was added and stirring continued for 10 min. A solution of benzophenone (9.1 g, 0.049 mol, 1.02 eq) and 4-hydroxybenzophenone (9.5 g, 0.048 mol, 1 eq) in THF (40 mL) was added slowly and the resulting mixture was heated to reflux for 20 hours (or until the carbonyl compounds were consumed, monitored by TLC). The reaction was quenched with 10% aqueous K_2CO_3 solution and taken up with dichloromethane. The organic layer was collected and concentrated. The crude was dissolved in chloroform (some tetrahydrofuran was added to enhance solubility) and adsorbed on silica-gel. Column chromatography (eluting with chloroform, product R_f = 0.24) afforded the title compound in a 30% yield.

δ_H (500 MHz, $CDCl_3$) 7.13-7.07 (9H, m, $\underline{H}_{Ar}$), 7.04-6.99 (6H, m, $\underline{H}_{Ar}$), 6.90-6.88 (2H, m, $\underline{H}_{Ar}$), 6.58-6.55 (2H, m, $\underline{H}_{Ar}$), 4.52 (1H, s, O$\underline{H}$).

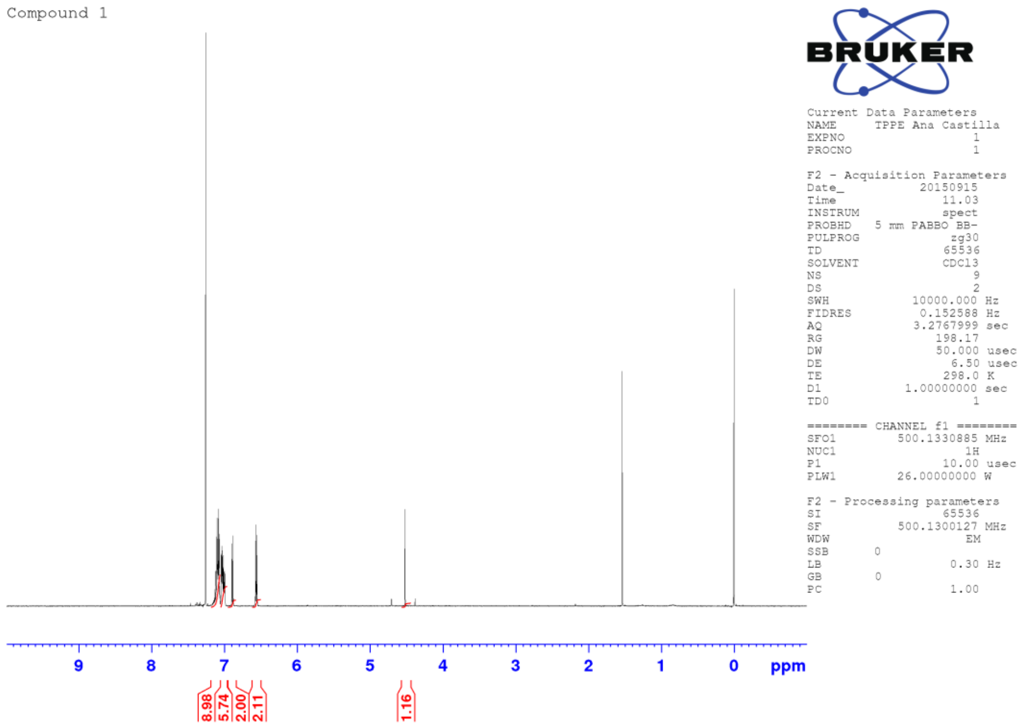

<u>*Tert*-butyl 2-[4-(1,2,2-triphenylethenyl)phenoxy]acetate (**1b**)</u>

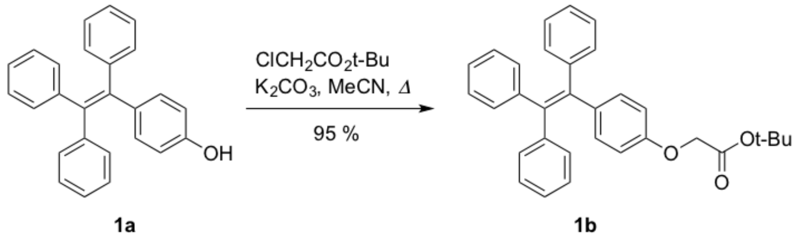

To a stirred solution of **1a** (4.56 g, 13mmol) and potassium carbonate (3.6g, 5 eq) in acetonitrile (100 mL) was added *tert*-butyl chloroacetate (1.87 mL, 1.1 eq). The solution was heated to reflux overnight at 100 °C. After this time, chloroform was added (100 mL), and the mixture was washed with water (4 × 100 mL). The organic phase was dried over magnesium sulfate and the solvent removed *in vacuo*. The crude product was purified by flash column chromatography, eluting with hexane/ethyl acetate 9:1, to give **1b** as an off-white solid (6 g, 95% yield).

δ_H (500 MHz, $CDCl_3$) 7.12-7.06 (9H, m, $\underline{H}_{Ar}$), 7.04-6.99 (6H, m, $\underline{H}_{Ar}$), 6.94-6.91 (2H, m, $\underline{H}_{Ar}$), 6.64-6.61 (2H, m, $\underline{H}_{Ar}$), 4.44 (2H, s, $C\underline{H}_2$), 1.46 (9H, s, $C(C\underline{H}_3)_3$).

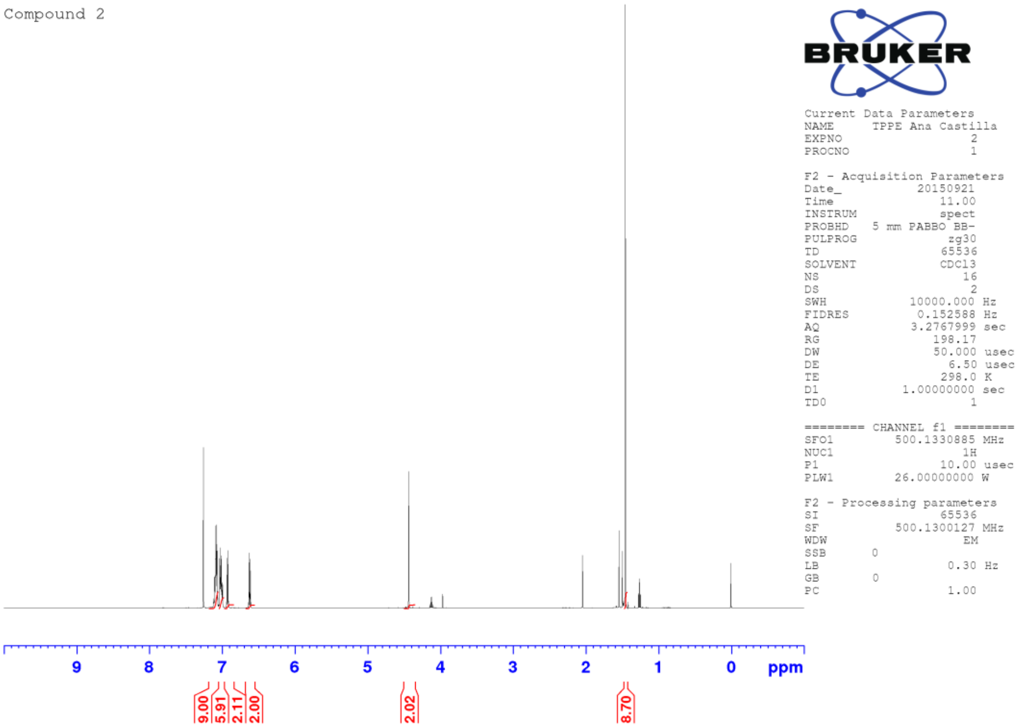

2-[4-(1,2,2-Triphenylethenyl)phenoxy]acetic acid (**1c**)

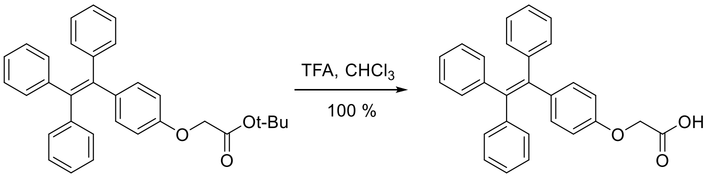

To a solution of **1b** in chloroform (20 mL), trifluoroacetic acid (10 mL) was added and the mixture was stirred overnight at room temperature. After this time diethyl ether was added to the reaction mixture and it was concentrated under reduce pressure. Dichloromethane was added and the mixture was concentrated again. This process of adding diethyl ether and dichloromethane and evaporating was repeated several times until the product was obtained as a white foam. The yield was near-quantitative.

δ_H (500 MHz, $CDCl_3$) 12.93 (1H, s, COOH), 7.17-7.05 (9H, m, H_{Ar}), 6.99-6.93 (6H, m, H_{Ar}), 6.87-6.85 (2H, m, H_{Ar}), 6.68-6.66 (2H, m, H_{Ar}), 4.58 (2H, s, CH_2).

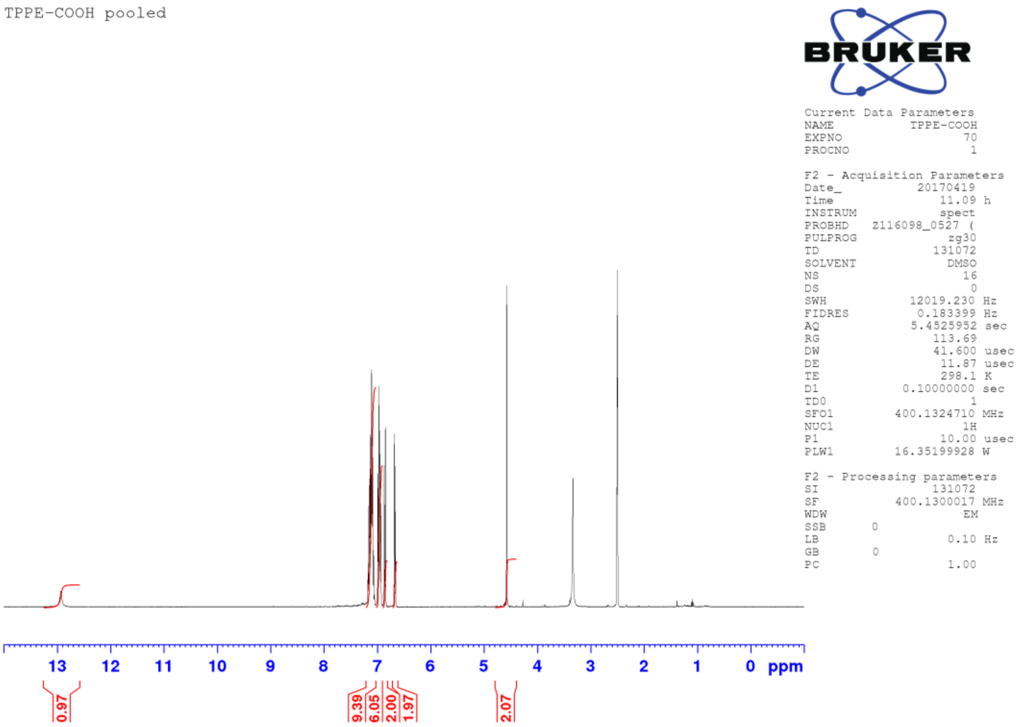

Ethyl (2*S*)-3-phenyl-2-[(2*S*)-3-phenyl-2-{2-[4-(1,2,2-triphenylethenyl)phenoxy]acetamido}propanamido]propanoate (**1d**)

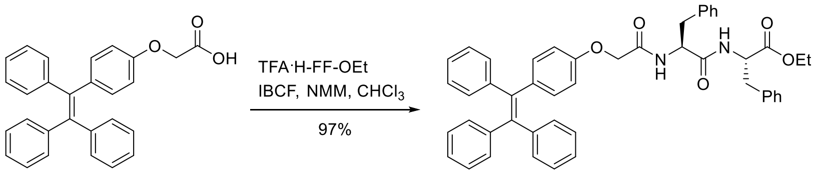

1c **1d**

To a solution of **1c** (965 mg, 2.37 mmol) in chloroform (20 mL) was added *iso*butyl chloroformate (1 eq, 308 μL), followed by *N*-methylmorpholine (1 eq, 260 μL). The mixture was stirred at ambient temperature for 15 minutes, after which time *bis*(*L*-phenylalanine) ethyl ester trifluoroacetate (1 eq, 1.08 g), and another portion of *N*-methylmorpholine (1 eq, 260 μL) were added, and the reaction mixture was stirred overnight. The reaction mixture was washed in turn with water, 1M hydrochloric acid, water again, and brine, dried ($MgSO_4$), filtered, and evaporated under reduced pressure. The required product was obtained as an off-white foam (1.67 g, 97%) in adequate purity for the next step. A small amount was purified *via* column chromatography (1:9 ethyl acetate/dichloromethane) to afford an analytical sample.

δ_H (400 MHz, DMSO-d_6) 8.57 (1H, d, *J* 7.48, N$\underline{H}$), 8.04 (1H, d, *J* 8.64, N$\underline{H}$), 7.28-7.07 (19H, m, $\underline{H}_{Ar}$), 6.99-6.93 (6H, m, $\underline{H}_{Ar}$), 6.82-6.79 (2H, m, $\underline{H}_{Ar}$), 6.55-6.53 (2H, m, $\underline{H}_{Ar}$), 4.63 (1H, td, 9.22, 4.36, C$\underline{H}^*$), 4.46 (1H, dd, *J* 14.43, 7.78, C$\underline{H}^*$), 4.32 (1H, d, *J* 15.29, OC$\underline{H}_a$H$_b$), 4.29 (1H, d, J 15.25, OCH$_a\underline{H}_b$), 4.03 (2H, q, *J* 7.10, C$\underline{H}_2$CH$_3$), 3.06-2.93 (3H, m, PhC$_a\underline{H}_2$ and PhC$_b\underline{H}_m$H$_n$), 2.78 (1H, dd, *J* 13.85, 9.80, PhC$_b$H$_m\underline{H}_n$), 1.09 (3H, t, *J* 7.10, CH$_2$C$\underline{H}_3$). δ_C (100 MHz, DMSO-d_6, not all aromatic carbon signals are resolved) 171.17, 170.91,

and 167.13 (C=O), 156.16, 143.39, 140.01, 139.79, 137.39, 136.94, 135.89, 131.84, 130.70, 130.63, 130.60, 129.17, 129.08, 128.21, 127.96, 127.87, 127.72, 126.54, 126.44, 126.40, 126.32, 126.23, and 113.86 (C_{Ar}), 66.46 (OCH_2), 60.51 (CH_2CH_3), 53.67 (CH^*), 52.95 (CH^*), 37.50 (PhC_bH_2), 36.66 (PhC_aH_2), 13.90 (CH_2CH_3). HRMS (ESI) m/z: $[M+Na]^+$ calcd for $C_{48}H_{44}N_2NaO_5$ 751.3142; found 751.3106.

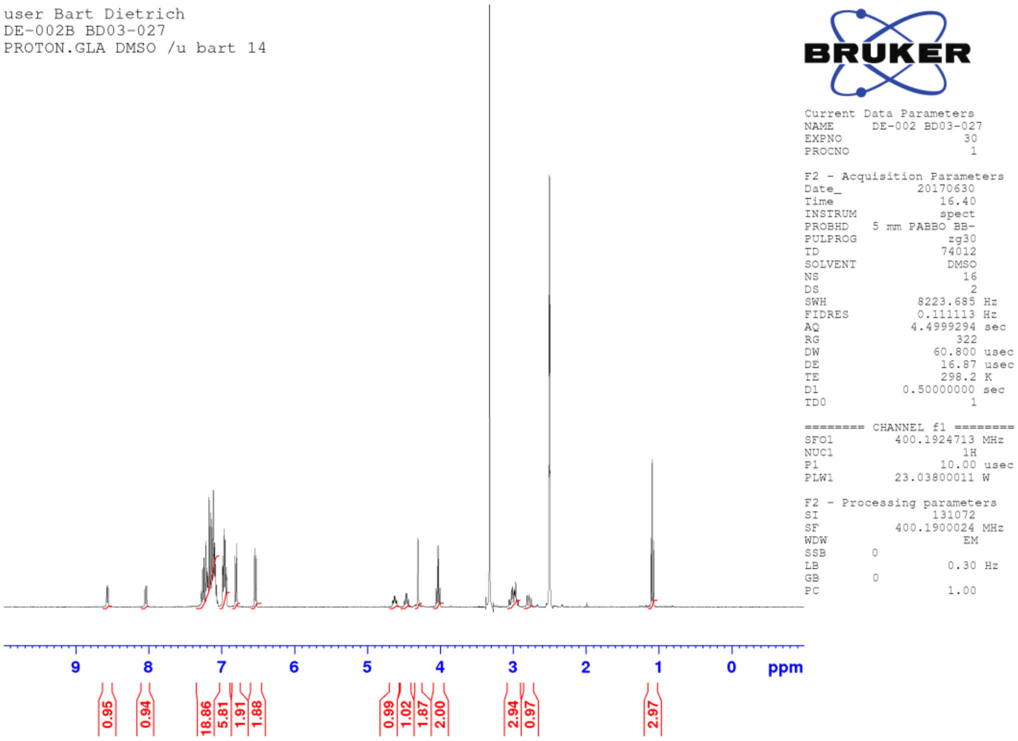

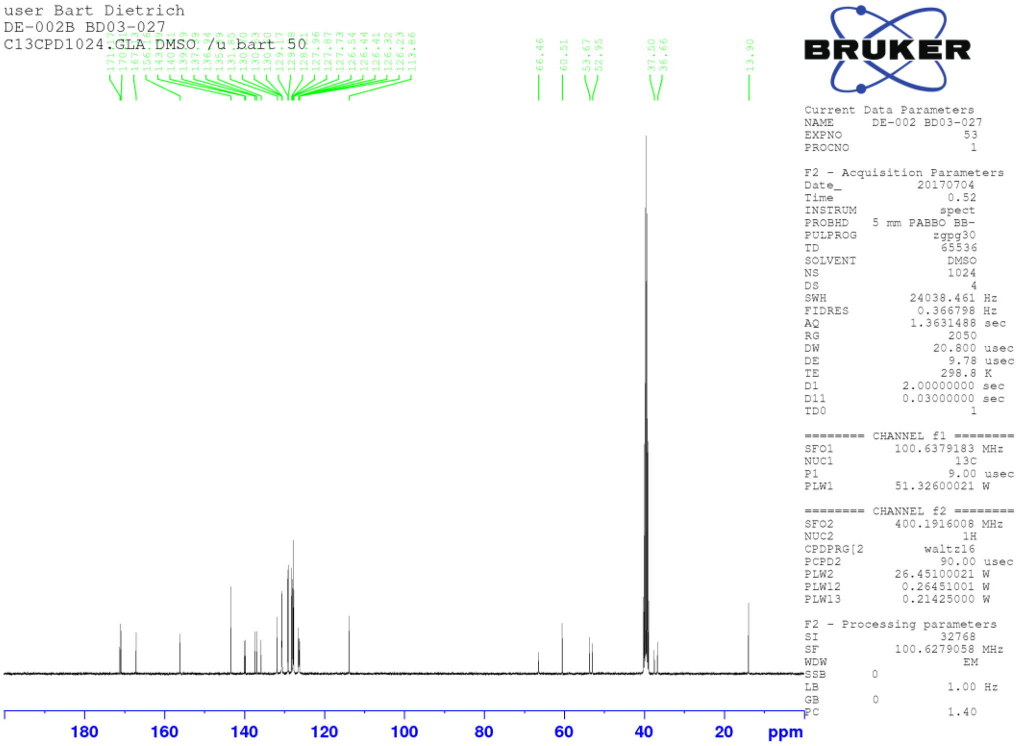

(2S)-3-Phenyl-2-[(2S)-3-phenyl-2-{2-[4-(1,2,2-triphenylethenyl)phenoxy]acetamido}propanamido]propanoic acid (1)

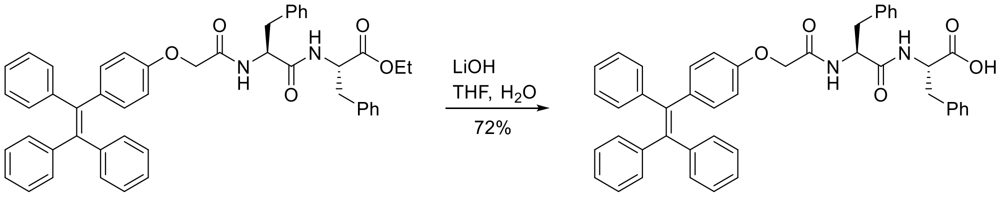

1d **1**

To a solution of **1d** (1.59 g, 2.18 mmol) in tetrahydrofuran (15 mL) was added a solution of lithium hydroxide (4 eq, 209 mg) in water (15 mL) and the mixture was stirred for 4 hours. After this time, TLC indicated the absence of starting material. The reaction mixture was concentrated to approximately half-volume, which caused a white solid to precipitate. This did not readily go back into solution on addition of water. The suspension was poured into 1M hydrochloric acid (*ca.* 200 mL) and stirred for 20 minutes. The precipitate was filtered off and washed with water in the filter. The resulting wet solid was dried by repeated azeotropic distillation with acetonitrile, then further dried at 70 °C under vacuum overnight. The title compound was thus obtained as a cream solid (1.10 g, 72%).

δ_H (400 MHz, DMSO-d_6) 12.78 (1H, br s, COOH), 8.38 (1H, d, *J* 7.88, NH), 7.99 (1H, d, *J* 8.68, NH), 7.27-7.05 (19H, m, H$_{Ar}$), 6.99-6.93 (6H, m, H$_{Ar}$), 6.82 (2H, m, H$_{Ar}$), 6.55-6.52 (2H, m, H$_{Ar}$), 4.64-4.57 (1H, m, CH*), 4.48-4.42 (1H, m, CH*), 4.30 (2H, s, OCH$_2$), 3.07 (1H, dd, *J* 13.86, 5.30, PhC$_a$H$_m$H$_n$), 2.99 (1H, dd, *J* 13.92, 4.28, PhC$_b$H$_m$H$_n$), 2.92 (1H, dd, *J* 13.82, 8.74, PhC$_a$H$_m$H$_n$), 2.78 (1H, dd, *J* 13.84, 9.72, PhC$_b$H$_m$H$_n$). δ_C (100 MHz, DMSO-d_6, not all aromatic carbon signals are resolved) 172.67, 170.78, and 167.10 (C=O), 156.16, 143.40, 140.02, 139.80, 137.44, 137.36, 135.90, 131.86, 130.71, 130.65, 130.62, 129.23, 129.11, 128.17,

127.95, 127.89, 127.74, 126.43, 126.34, 126.21, and 113.87 ($\underline{C}_{Ar}$), 66.48 ($O\underline{C}H_2$), 53.47 ($\underline{C}^*$), 52.99 ($\underline{C}^*$), 37.47 ($Ph\underline{C}_bH_2$), 36.65 ($Ph\underline{C}_aH_2$) HRMS (ESI) m/z: $[M+Na]^+$ calcd for $C_{46}H_{40}N_2NaO_5$ 723.2829; found 723.2806.

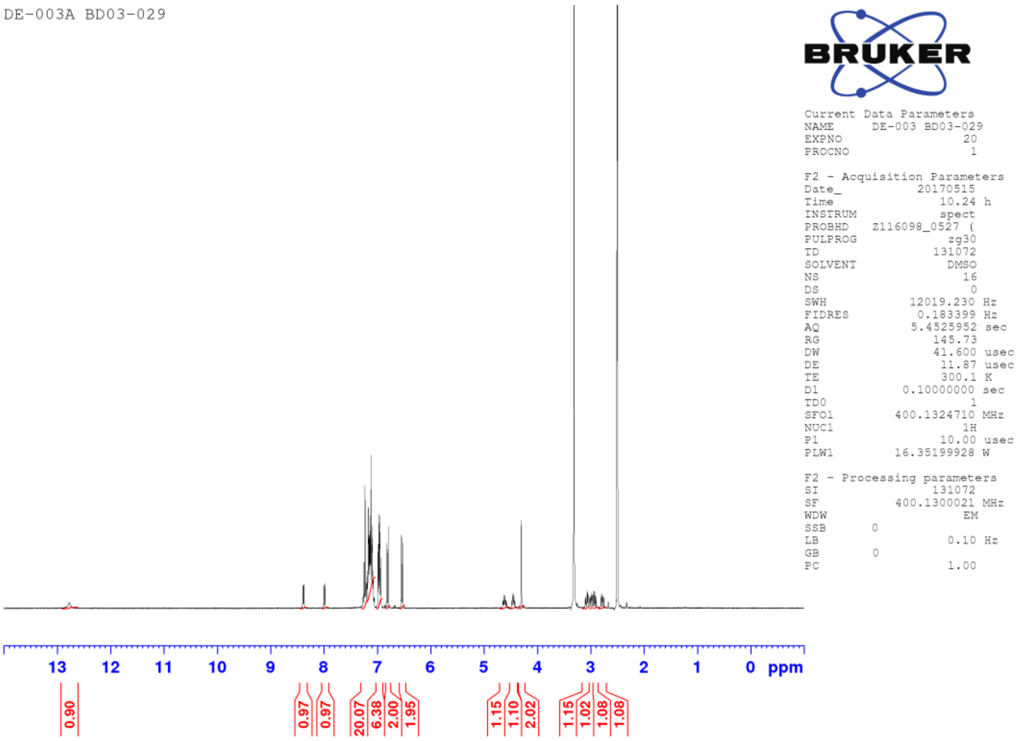

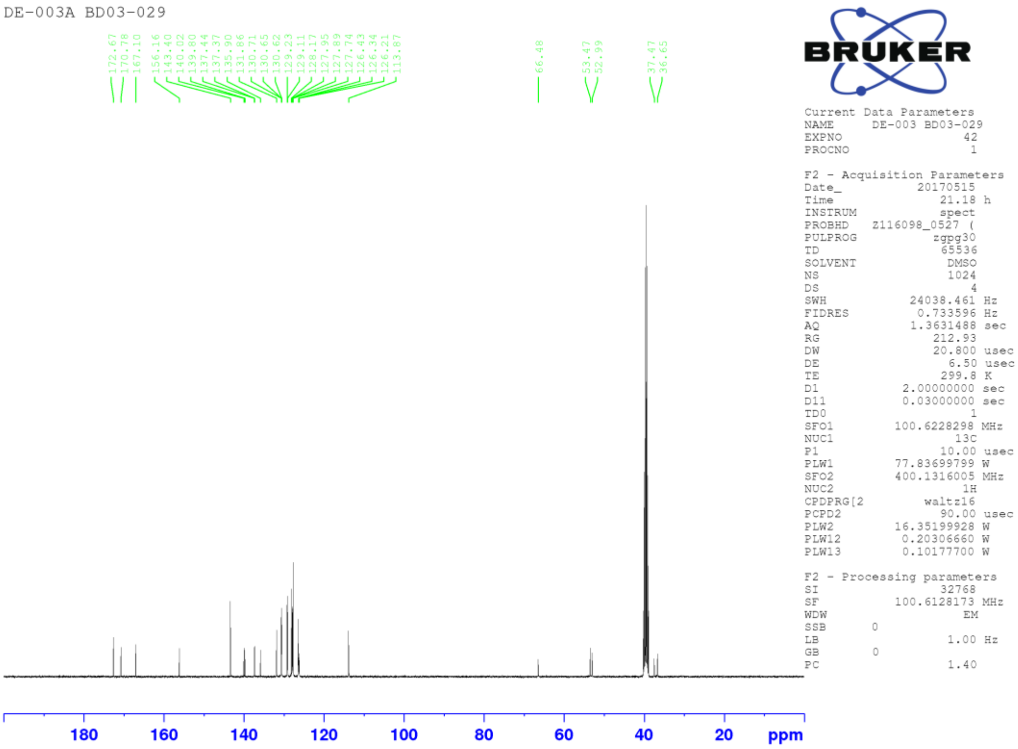

Supplementary Figures.

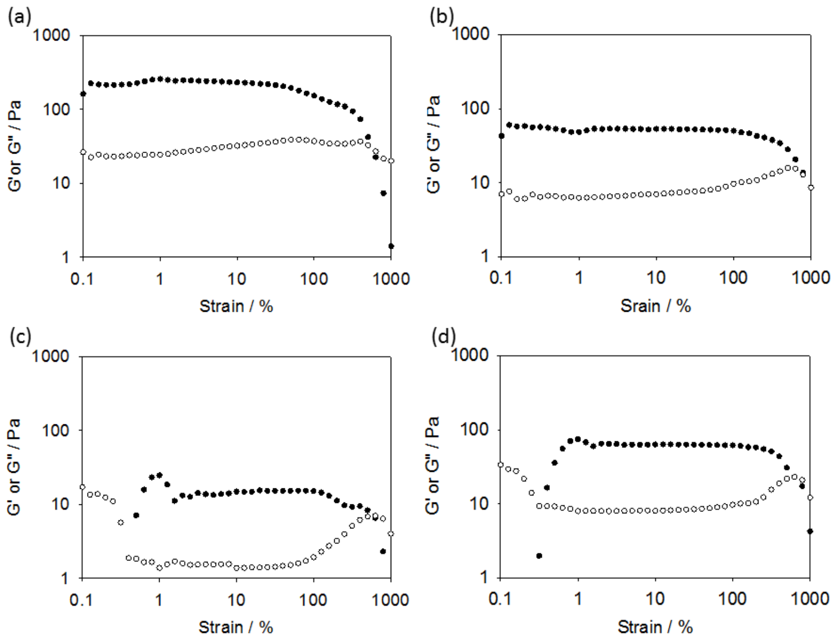

Figure S1. Strain sweeps for self-supporting samples formed by the addition of NaCl to solutions of **1** (a) 10 mg/mL; (b) 7.5 mg/mL; (c) 5 mg/mL; (d) 2.5 mg/mL; in all cases, full symbols represent G′ and open symbols represent G″ and a constant ratio of NaCl: **1** of 1:8 was used.

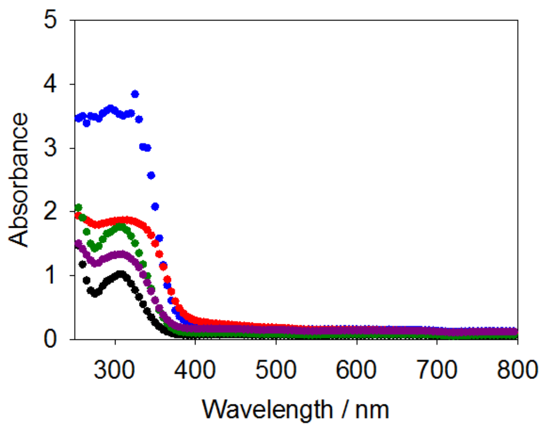

Figure S2. UV-Vis spectra for solutions of **1** at 5 mg/mL. Black data are for the as-prepared solution. Dark pink data are for a solution after heating and cooling. Red data are for a solution to which $CaCl_2$ has been added. Green data are for a solution to which NaCl has been added. Blue data are for a solution to which GdL has been added. All data were collected in a 0.1 mm cuvette.

References

1. Duan, X.-F.; Zeng, J.; Lu, J.-W.; Zhang, Z.-B., Insights into the General and Efficient Cross McMurry Reactions between Ketones. *J. Org. Chem.* **2006,** *71* (26), 9873-9876.

2. Wang, H.; Huang, Y.; Zhao, X.; Gong, W.; Wang, Y.; Cheng, Y., A novel aggregation-induced emission based fluorescent probe for an angiotensin converting enzyme (ACE) assay and inhibitor screening. *Chem. Commun.* **2014,** *50*, 15075-15078.
